# System-Wide Analysis of Protein Acetylation and Ubiquitination Reveals a Diversified Regulation in Human Cancer Cells

**DOI:** 10.3390/biom10030411

**Published:** 2020-03-06

**Authors:** Hiroko Kozuka-Hata, Aya Kitamura, Tomoko Hiroki, Aiko Aizawa, Kouhei Tsumoto, Jun-ichiro Inoue, Masaaki Oyama

**Affiliations:** 1 Medical Proteomics Laboratory, The Institute of Medical Science, The University of Tokyo, 4-6-1, Shirokanedai, Minato-ku, Tokyo 108-8639, Japan; a-kitamr@ims.u-tokyo.ac.jp (A.K.); t-hiroki@ims.u-tokyo.ac.jp (T.H.); a-aizawa@ims.u-tokyo.ac.jp (A.A.); tsumoto@ims.u-tokyo.ac.jp (K.T.); jun-i@ims.u-tokyo.ac.jp (J.-i.I.); 2 Department of Bioengineering, Graduate School of Engineering, The University of Tokyo, 7-3-1, Hongo, Bunkyo-ku, Tokyo 113-8656, Japan; 3 Department of Cancer Biology, The Institute of Medical Science, The University of Tokyo, 4-6-1, Shirokanedai, Minato-ku, Tokyo 108-8639, Japan

**Keywords:** proteomics, cancer, ubiquitination, acetylation, bioinformatics

## Abstract

Post-translational modifications are known to be widely involved in the regulation of various biological processes, through the extensive diversification of each protein function at the cellular network level. In order to unveil the system-wide function of the protein lysine modification in cancer cell signaling, we performed global acetylation and ubiquitination proteome analyses of human cancer cells, based on high-resolution nanoflow liquid chromatography–tandem mass spectrometry, in combination with the efficient biochemical enrichment of target modified peptides. Our large-scale proteomic analysis enabled us to identify more than 5000 kinds of ubiquitinated sites and 1600 kinds of acetylated sites, from representative human cancer cell lines, leading to the identification of approximately 900 novel lysine modification sites in total. Very interestingly, 236 lysine residues derived from 141 proteins were found to be modified with both ubiquitination and acetylation. As a consequence of the subsequent motif extraction analyses, glutamic acid (E) was found to be highly enriched at the position (−1) for the lysine acetylation sites, whereas the same amino acid was relatively dispersed along the neighboring residues of the lysine ubiquitination sites. Our pathway analysis also indicated that the protein translational control pathways, such as the eukaryotic initiation factor 2 (EIF2) and the ubiquitin signaling pathways, were highly enriched in both of the acetylation and ubiquitination proteome data at the network level. This report provides the first integrative description of the protein acetylation and ubiquitination-oriented systematic regulation in human cancer cells.

## 1. Introduction

Post-translational modifications (PTMs), such as phosphorylation, acetylation, and ubiquitination, are widely known to play various important roles in cellular signaling, and more than 1000 kinds of PTMs regarding eukaryotes and prokaryotes have been registered with Unimod, a comprehensive database of protein modifications for mass spectrometry [1]. Recent technological advances in mass-spectrometry-based proteomics, in combination with micro-purification techniques for each PTM peptide, have enabled us to perform a comprehensive identification and quantification of PTMs [2].

Protein phosphorylation is recognized as one of the most intensively studied PTMs, and it regulates a variety of key cellular processes by transmitting diverse signals through the enzymatic reaction of kinases/phosphatases and their substrates [3]. Previous extensive phosphoproteomic studies under various experimental conditions allowed us to uncover and record hundreds of thousands of phosphorylation sites in a public database, such as PhosphoSitePlus [4], and some computational platforms have been developed to analyze phosphorylation-dependent protein–protein interaction networks at the system level [5,6]. Protein lysine acetylation is also known to play essential roles in the transcriptional regulation by hub protein molecules, such as histone and p53, through the coordinated function of acetyltransferases and deacetylases [7]. In addition, the lysine ubiquitination widely contributes to the transmission of protein degradation signals, as well as the cell-cycle progression and DNA repair [8].

Based on the successful establishment of PTM-specific antibodies for the efficient enrichment of target modified peptides, recent technological advances in mass-spectrometry-based proteomics have led us to identify thousands of lysine acetylation and ubiquitination peptides, in a comprehensive and unbiased manner [9,10,11,12]. In this study, we performed a high-resolution mass-spectrometry-based comprehensive analysis of protein acetylation and ubiquitination, regarding various human cancer cells, to systematically characterize the network-wide signaling potentials coordinated by differential lysine modifications.

## 2. Materials and Methods

### 2.1. Sample Preparation for Mass Spectrometry

For this study, we used 13 human cancer cell lines from 6 different tissues as indicated below: KG-1-C, U251, T98G, U87, GB2 (brain), NCI-H3255, NCI-H1650, NCI-H441 (lung), MCF7, HCC1954 (breast), AGS (stomach), A431 (skin), and HeLa (cervix) cells. GB2 cells were originally established from the tumor tissues classified as primary glioblastoma in the University of Tokyo Hospital, with informed consent, and they were approved by the Research Ethics Committee at the Institute of Medical Science of the University of Tokyo, as previously reported [6,13]. Cancer cell lysates were centrifuged for 30 min at 15,000 rpm and the obtained supernatants were digested overnight, using a sequencing-grade modified trypsin (Promega, Madison, WI, USA), according to a previous study [13]. The acetylated peptides were enriched using the Acetyl-lysine motif kit (Cell Signaling Technology, Danvers, MA, USA), whereas the tryptic peptides with ubiquitination-derived diglycine remnants were purified using the Ubiquitin remnant motif kit (Cell Signaling Technology, Danvers, MA, USA), as recommended by the manufacturer. The enriched peptides were then desalted using ZipTip C18 (Millipore, Billerica, MA, USA), and evaporated down to a volume of up to 10 µL using a vacuum concentrator.

### 2.2. Shotgun Proteomic Analysis by a Nanoflow Liquid Chromatography–Tandem Mass Spectrometry (nanoLC-MS/MS) System

The peptide samples were analyzed using a Dina-2A nanoflow liquid chromatography (LC) system (KYA Technologies, Tokyo, Japan), coupled with an LTQ-Orbitrap Velos mass spectrometer (Thermo Fisher Scientific, Bremen, Germany), as previously reported [13]. Peptides were injected into a 75-µM reversed-phase C18 column at a flow rate of 10 µL/min, and eluted with a linear gradient of solvent A (2% acetonitrile and 0.1% formic acid in H_2_O) to solvent B (40% acetonitrile and 0.1% formic acid in H_2_O), at 300 nL/min. The separated peptides were sequentially sprayed from a nanoelectrospray ion source (KYA Technologies, Tokyo, Japan), and analyzed by the collision induced dissociation (CID) method. The mass spectra were acquired in a data-dependent mode, by switching automatically the MS and MS/MS acquisition modes. All full-scan MS spectra in the range from *m*/*z* 300 to 1600 were acquired in the FT-MS part of the mass spectrometer, with a target value of 1,000,000 and a resolution of 100,000 at *m*/*z* 400. The 20 most intense ions that satisfied an ion selection threshold above 2000 were fragmented in the linear ion trap, with a normalized collision energy of 35% for an activation time of 10 ms. For an accurate mass measurement, the Orbitrap analyzer was operated with the “lock mass” option, using polydimethylcyclosiloxane (*m*/*z* = 445.120025) and bis(2-ethylhexyl) phthalate ions (*m*/*z* = 391.284286).

### 2.3. Large-Scale Identification of Lysine Ubiquitination and Acetylation Sites

The protein identification was performed by searching the MS and MS/MS data against the RefSeq (National Center for Biotechnology Information) human protein database, using Mascot (Matrix Science, London, UK). The carbamidomethylation of cysteine was set as a fixed modification, whereas the oxidation of methionine, protein N-terminal diglycine/acetylation, pyro-glutamination for N-terminal glutamine, and diglycine/acetylation of lysine were set as variable modifications. Trypsin was defined as a proteolytic enzyme, and a maximum of three or six missed cleavages were allowed, to identify the ubiquitination and acetylation sites in our database search. The mass tolerance was set to 3 parts per million (ppm) for peptide masses and 0.8 Da for MS/MS peaks, respectively. In the process of peptide identification, we conducted a decoy database search by Mascot, and applied a filter to satisfy a false positive rate lower than 1%.

### 2.4. Flanking Amino Acid Sequence Analysis

For the representation of position weight matrices (PWMs) for (−9) to (+9) amino acid residues surrounding all of the identified lysine ubiquitination and acetylation sites, the probability of the observed amino acid residues, at each position on the flanking sequences of the lysine modification sites, was normalized by the abundance ratio of each amino acid in the RefSeq human protein database. The visualization of the statistically extracted sequence motifs, based on our large-scale lysine modification proteome data, was also performed using motif-X [14,15].

### 2.5. Pathway Analysis

The computational analysis for the statistical extraction of canonical pathways was performed using Ingenuity Pathway Analysis (IPA, QIAGEN, Redwood City, CA, USA) [16]. The proteins modified with the ubiquitination and/or acetylation were uploaded into the IPA software (version 2018-2019), and the top canonical pathways associated with the uploaded proteins were listed along with the *p*-values calculated using a right tailed Fisher’s exact test [17].

## 3. Results and Discussion

### 3.1. Large-Scale Identification of Protein Acetylation and Ubiquitination Sites Reveals Cell-Type Dependent Qualitative and Quantitative Diversities

**Figure 1:**
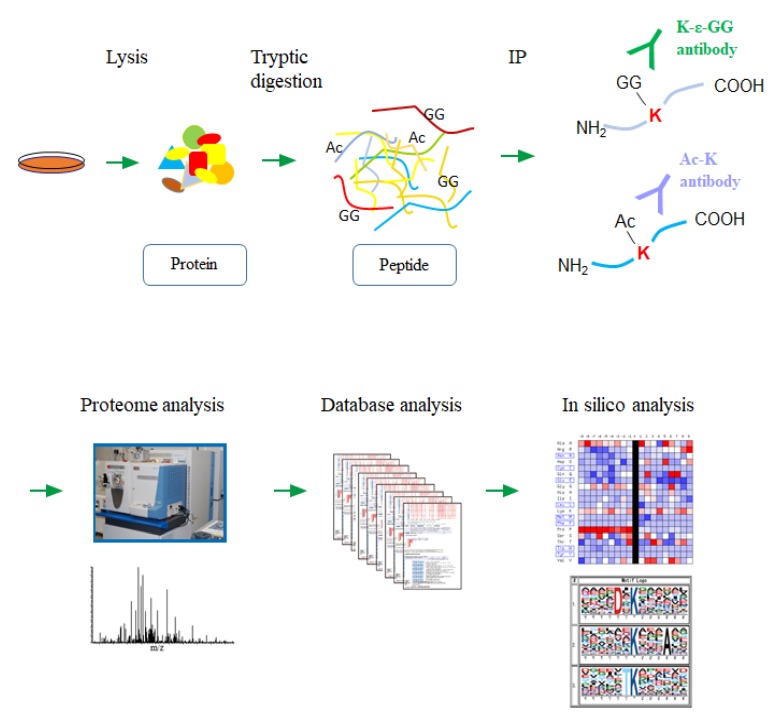
A schematic workflow for the large-scale lysine modification proteome analysis. Each cancer cell lysate was digested with trypsin, and the lysine-modified peptides were immunoprecipitated using the specific antibodies. Regarding the ubiquitination, the enzymatically generated diglycine remnants on the side-chain of modified lysine residues (K-ε-GG) were recognized by the corresponding antibody. The enriched peptides were then analyzed by the high-resolution nanoLC-MS/MS system, followed by an integrative computational analysis.

In order to grasp the network-wide status of the protein lysine modification in various types of human cancer cells, we performed a double-edged proteomic analysis of the lysine ubiquitination and acetylation by a high-resolution nanoLC-MS/MS coupled with an antibody-based enrichment of the corresponding lysine-modified peptides from each cell lysate (Figure 1).

**Figure 2:**
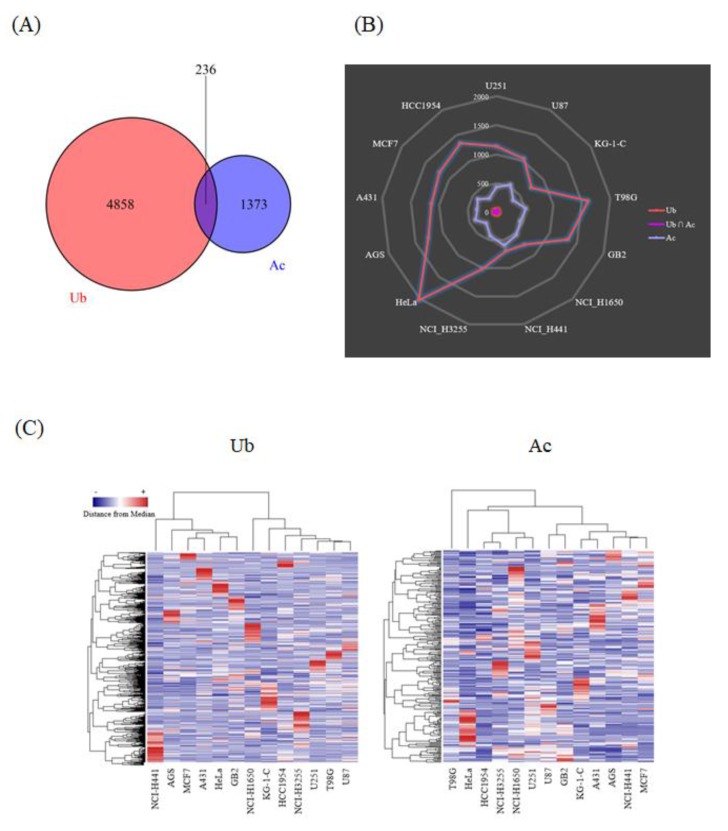
Summary of the ubiquitination and acetylation proteome data on human cancer cells. (**A**) The Venn diagram for the total number of the identified peptides with ubiquitinated/acetylated amino acid residues from all of the cancer cell lines analyzed in this study. (**B**) The radar chart for the comparative distribution of the ubiquitinated and/or acetylated peptides detected from each human cancer cell line. (**C**) The heat map for the hierarchical clustering of the ubiquitination and acetylation proteome data on thirteen human cancer cell lines. The clustered columns on the *y*-axis indicate the respective modification sites identified in our high-resolution proteomic analysis.

Thousands of acetylated and ubiquitination-derived diglycine-remnant peptides were detected from each of the human cancer cell lines, leading to the identification of more than 5000 ubiquitination sites and 1600 acetylation sites, including 236 lysine residues dually modified with acetylation and ubiquitination (Figure 2A). Very interestingly, our large-scale proteomic data revealed that the cellular tumor antigen p53, one of the most critical transcription factors involved in cancer cell signaling [18], was modified with these two types of lysine modifications (Supplementary Table S1 and S2). The ubiquitinated p53 peptides were detected from T98G, MCF7, AGS, and HeLa cells, whereas the acetylated p53-derived peptides were identified from U251 cells. One of the well-characterized lysine sites on the p53 protein sequence, Lys382, was found to be acetylated in our proteomic study, as previously reported [18]. Our results also showed that the relative frequency of ubiquitinated and acetylated peptide counts dramatically varied among the investigated cancer cell lines (Figure 2B), and that the hierarchical clustering of the whole protein acetylation and ubiquitination datasets clearly indicated their cell-type dependent characteristics (Figure 2C). Thus, our integrative ubiquitination and acetylation proteome data on human cancer cells unveiled qualitative and quantitative diversities regarding lysine-modification-related cellular networks.

### 3.2. Statistical Enrichment Analysis of Protein Acetylation and Ubiquitination Datasets Highlights Lysine-Modification-Related Flanking Amino Acid Sequences and Core Signaling Pathways in Human Cancer Cells

**Figure 3:**
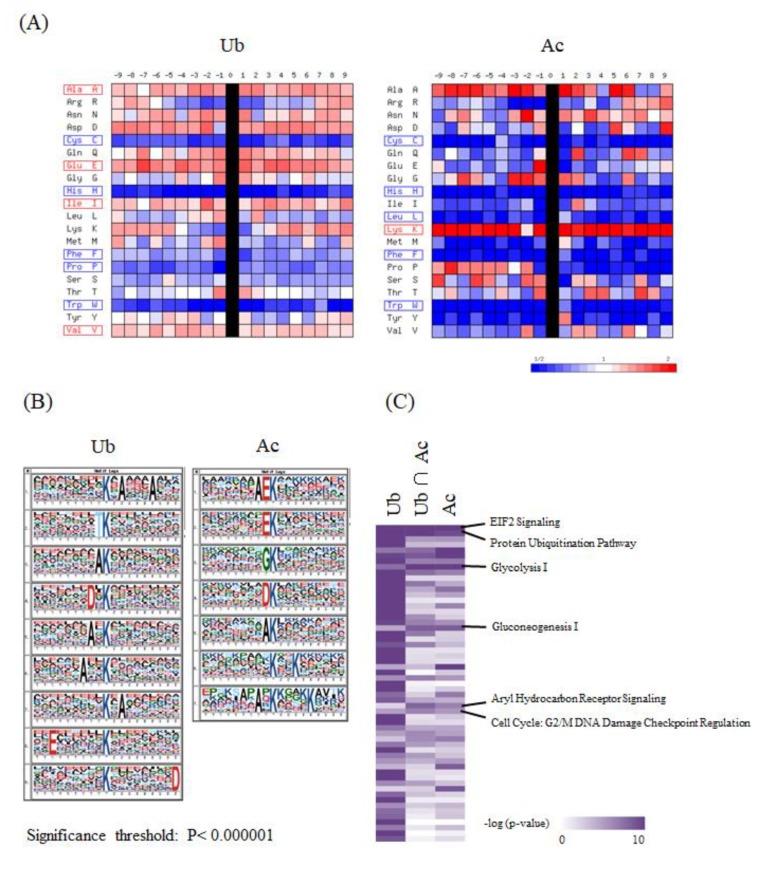

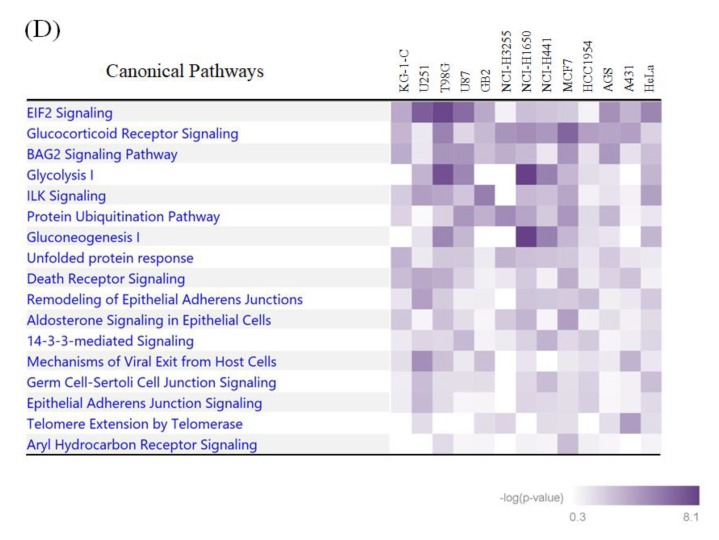
The integrative bioinformatic analysis of the large-scale protein ubiquitination and acetylation data. (**A**) The visualization of position weight matrices (PWMs) for the flanking amino acid residues of the ubiquitination and acetylation sites identified in our large-scale proteomic measurements. The probability of the observed amino acid residues at each flanking position was normalized by the abundance ratio of each amino acid in the NCBI RefSeq human protein database. (**B**) The visualization of statistically extracted sequence motifs based on our large-scale lysine modification proteome data. The sequence motifs surrounding the lysine modification residues were represented as sequence logos by the motif-x algorithm. (**C**) The heatmap for the canonical pathways defined by the Ingenuity Pathway Analysis (IPA). The representative canonical pathways are indicated at the right side of the clustered data. (**D**) The IPA-based canonical pathway analysis of the dually modified proteins identified from each cancer cell line.

In order to characterize the system-wide functionality mediated by the lysine acetylation and ubiquitination, we performed further integrative bioinformatic analyses of the large-scale ubiquitination and acetylation datasets obtained from our proteomic measurements. First, we analyzed the statistical features of the flanking amino acid sequences of the lysine modification sites, in comparison with all of the protein lysine residues stored in the public human protein database. The visualization of PWMs for the (−9) to (+9) amino acid residues from the identified ubiquitination and acetylation sites indicated that the frequency of cysteine (C), histidine (H), and tryptophan (W) was reduced along the overall positions for both of these two lysine modification sites, whereas lysine (K) was enriched for the lysine acetylation sites (Figure 3A). Furthermore, the sequence logos extracted by the motif-X algorithm [14,15] revealed that glutamic acid (E) was highly enriched at the position (–1) for the lysine acetylation sites, which was consistent with the previous analysis of the acetylation proteome data on rat cytoplasmic and ER-Golgi proteins [12]. In contrast, the same amino acid was relatively dispersed along the neighboring residues of the lysine ubiquitination sites (Figure 3B).

We next performed an IPA-based pathway enrichment analysis to statistically extract core biological pathways governed by these two types of protein modifications. A heat map visualization of the canonical pathways based on the Ingenuity Knowledge Base [16] showed that the eukaryotic initiation factor 2 (EIF2) signaling and protein ubiquitination pathways were highly enriched in the acetylation proteome data as well as in the ubiquitination data (Figure 3C). Our analysis also indicated that metabolic pathways, such as glycolysis and gluconeogenesis, were prominently correlated with acetylation rather than ubiquitination, whereas the aryl hydrocarbon receptor signaling and the cell cycle were found to be preferentially involved in dual modification by ubiquitination and acetylation. Regarding the identified proteins with dually modified lysine residues listed in Supplementary Table S3, we further performed an IPA-based pathway analysis, and found that the EIF2 signaling pathway was relatively enriched in brain-derived cancer cell lines (Figure 3D).

## 4. Conclusions

Our large-scale analysis of protein acetylation and ubiquitination based on shotgun nanoLC-MS/MS detection led to the identification of more than 6000 lysine modification sites from human cancer cells. This study reports the first integrative description of the lysine modification proteome, regarding cancer, and it uncovers the cell type-dependent acetylation and ubiquitination diversity at the network level. A further computational analysis, based on the accumulating lysine modification proteome data, in combination with the machine-learning-oriented prediction of lysine modification sites [19,20], will accelerate the extensive dissection of the protein lysine-modification-mediated regulatory networks at the system level.

## Supplementary Materials

The following are available online at https://www.mdpi.com/2218-273X/10/3/411/s1, Supplementary Table S1: The proteomic data on the ubiquitinated peptide counts regarding thirteen human cancer cell lines, Supplementary Table S2: The proteomic data on the acetylated peptide counts regarding thirteen human cancer cell lines. Supplementary Table S3: The list of the proteins with dually modified lysine residues identified from each cancer cell line.
